# Pyrexia and Open Air

**Published:** 1902-06-28

**Authors:** 


### PYREXIA AND OPEN AIR.

Speaking recently on the subject of the treatment of pulmonary tuberculosis by hygiene, Dr. Theodore Williams made some remarks concerning the use of open air in pyrexial cases which probably have an application in regard to other forms of fever besides that which is associated with phthisis. He says that one of the great advantages of the open-air treatment is that it is suited not only to cases of early and limited pulmonary tuberculosis which, as we all know, can be arrested by various measures, but that its benefits are also well shown in cases of extensive tuberculisation with cavities, irritable or quiescent, and above all in pyrexial cases where, owing to the pulmonary changes, the pyrexia is often very persistent. In such patients after all the antipyretics, pharmacopœial and extra-pharmacopœial, have been exhausted, it is found that exposure, on a bed or on a light couch, by day and by night, to the pure air in a shelter or verandah, will in time prove effective and that the pyrexia will gradually subside. How fresh air at a comparatively low temperature affects the thermotaxic and thermogenic nerve centres, if there be such things, and thus reduces fever it may not be easy to explain, and Dr. Williams confesses that he cannot say whether this is the result of its soothing the nerve centres or stimulating and finally exhausting the combustive process, but the fact he has noted in many instances. "I have known patients," he says, "with active tuberculous disease, accompanied by afternoon pyrexia which had lasted for months, placed on couches in shelters, and I have observed that in most of these cases the pyrexia has subsided after some weeks in the open air." Many of these patients were not in sanatoria but in their own gardens under the supervision of a medical man or a nurse. The open-air life at first, and especially in pyrexial cases, causes a feeling of chilliness, but this soon passes off with proper clothing, and particularly if the patient remains lying down with the head and thorax only sufficiently raised to render expectoration easy. Patients who are chilly in the sitting position can often stand an extraordinarily low temperature when lying down, from the more equalising effect of this position upon the circulation. Of course, draughts, and as much as possible wind, must be avoided, which can be successfully accomplished with suitable windows and properly constructed liegehalles or revolving shelters. Pyrexia should first be treated by the patient lying in bed with open windows, and if the temperature does not fall the bed should be moved on to a terrace or covered balcony protected from rain but freely open to the air. We cannot but feel that a line of treatment which has shown itself so beneficial in phthisis ought to have a far wider applicability, and especially in the management of septic cases, for it must be remembered that, according to all recent teaching, the pyrexia of phthisis is not an essentially tuberculous process, but is largely the result of the superadded, often no doubt septic infections with which the malady has become complicated.

